# Quantum++: A modern C++ quantum computing library

**DOI:** 10.1371/journal.pone.0208073

**Published:** 2018-12-10

**Authors:** Vlad Gheorghiu

**Affiliations:** 1 softwareQ Inc., Kitchener ON, Canada; 2 Institute for Quantum Computing, University of Waterloo, Waterloo ON, Canada; University of the Basque Country, SPAIN

## Abstract

Quantum++ is a modern general-purpose multi-threaded quantum computing library written in C++11 and composed solely of header files. The library is not restricted to qubit systems or specific quantum information processing tasks, being capable of simulating arbitrary quantum processes. The main design factors taken in consideration were the ease of use, portability, and performance. The library’s simulation capabilities are only restricted by the amount of available physical memory. On a typical machine (Intel i5 8Gb RAM) Quantum++ can successfully simulate the evolution of 25 qubits in a pure state or of 12 qubits in a mixed state reasonably fast. The library also includes support for classical reversible logic, being able to simulate classical reversible operations on billions of bits. This latter feature may be useful in testing quantum circuits composed solely of Toffoli gates, such as certain arithmetic circuits.

## 1 Introduction

Quantum computing is a disruptive technology that promises great benefits for a plethora of applications, ranging from medicine and chemistry to machine learning and simulation of physical systems. However, today’s most advanced quantum computers are not yet large enough for performing universal quantum computation, hence their applicability is still limited. Being able to simulate small sized quantum computers is therefore of paramount importance, as it allows the scientist or engineer to understand better the results she or he would expect from a quantum machine of similar size, as well as providing a better understanding of quantum computing itself. Below we describe a quantum computing library that can be used in research or exploratory work in quantum information and computation.

Quantum++, available online at https://github.com/vsoftco/qpp, is a C++11 general purpose quantum computing library, composed solely of header files. It uses the Eigen 3 linear algebra library and, if available, the OpenMP multi-processing library. For additional Eigen 3 documentation see http://eigen.tuxfamily.org/dox/. For a simple Eigen 3 quick ASCII reference see http://eigen.tuxfamily.org/dox/AsciiQuickReference.txt.

The simulator defines a large collection of (template) quantum computing related functions and a few useful classes. The main data types are complex vectors and complex matrices, which we will describe below. Most functions operate on such vectors/matrices passed by value and always return the result by value, without ever mutating their arguments. The design is inspired from functional programming, where functions do not mutate their arguments and do not have side effects. Those design choices make the library ideal to use or integrate in multi-processing frameworks. Collection of objects are implemented via the standard library container std::vector<>, instantiated accordingly.

We decided to avoid using a complicated class hierarchy and focus on a functional style-like approach, as we believe the latter is more suitable for a relatively small API and allows the user to focus on the quantum algorithm design rather than on object-oriented design. In addition, there is absolutely no need for explicit memory allocations or usage of (raw) pointers. All allocations, initializations and release of resources are performed by the library, hence the user is not at risk of forgetting to de-allocate memory, use un-initialized objects, or overflowing buffers, which are the most common, dangerous and hard to diagnose mistakes in the world of C and C++ programming.

Although there are many available quantum computing libraries/simulators written in various programming languages, see [1] for a comprehensive list, what makes Quantum++ different is the ease of use, portability and high performance. The library is not restricted to specific quantum information tasks, but it is intended to be multi-purpose and capable of simulating arbitrary quantum processes. We have chosen the C++ programming language (standard C++11) in implementing the library as it is by now a mature standard, fully (or almost fully) implemented by the most common compilers, and highly portable.

Other unique features of Quantum++ include the ability of simulating classical reversible networks up to billions of bits (this feature may be useful in testing quantum circuits composed solely of Toffoli gates, such as certain arithmetic circuits in e.g. [2]), strong multi-threading abilities, as well as built-in support for higher dimensional systems (qudits) that allows treating qubits and qudits on the same footing.

In the reminder of this manuscript we describe the main features of the library, “in a nutshell” fashion, via a series of simple examples. We assume that the reader is familiar with the basic concepts of quantum mechanics/quantum information. For a comprehensive introduction to the latter see e.g. [3]. This document is not intended to be a comprehensive documentation, but only a brief introduction to the library and its main features. For a detailed reference see the manual available as a .pdf file in ./doc/refman.pdf. For detailed installation instructions as well as for additional information regarding the library see the Wiki page at https://github.com/vsoftco/qpp/wiki. If you are interesting in contributing, or for any comments or suggestions, please contact me.

Quantum++ is free software: you can redistribute it and/or modify it under the terms of the MIT License https://opensource.org/licenses/MIT.

## 2 Installation

To get started with Quantum++, first install the Eigen 3 library from http://eigen.tuxfamily.org into your home directory, as $HOME/eigen. Here we implicitly assume that you use a UNIX-like system, although everything should translate into Windows as well, with slight modifications. You can change the name of the directory, but in the current document we will use $HOME/eigen as the location of the Eigen 3 library. Next, download the Quantum++ library from https://github.com/vsoftco/qpp/ and unzip it into the home directory as $HOME/qpp. Finally, make sure that your compiler supports C++11 and preferably OpenMP. For a compiler we recommend g++ version 5.0 or later or clang version 3.7 or later (previous versions of clang do not support OpenMP).

We next build a simple minimal example to test that the installation was successful. Create a directory called $HOME/testing, and inside it create the file minimal.cpp, with the content listed in Listing 1. A verbatim copy of the above program is also available at $HOME/qpp/examples/minimal.cpp.

**Listing 1. Minimal example**

1 // Minimal example//

2 // Source: ./examples/minimal.cpp

3 #include <iostream>

4

5 #include “qpp.h”

6

7 int main() {

8  using namespace qpp;

9  std::cout << “Hello Quantum++!\nThis is the |0> state:\n”;

10  std::cout << disp(st.z0) << ‘\n’;

11 }

Next, compile the file using a C++11 compliant compiler. In the following, we assume that you use g++, but the building instructions are similar for other compilers. From the directory $HOME/testing type

g++ -std = c++11 -O3 -Wall -Wextra -pedantic -isystem $HOME/eigen\

-I $HOME/qpp/include minimal.cpp -o minimal

Your compile command may differ from the above, depending on the C++ compiler and operating system. If everything went fine, the above command should build an executable minimal in $HOME/testing, which can be run by typing ./minimal. The output should be similar to the following:

**Listing 1 output**

Hello Quantum++!

This is the |0> state:

1

0

In line 5 of Listing 1 we include the main header file of the library qpp.h This header file includes all other necessary internal Quantum++ header files. In line 10 we display the state |0〉 represented by the singleton st.z0 in a nice format using the display manipulator disp().

## 3 Data types, constants and global objects

All header files of Quantum++ are located inside the ./include directory. All functions, classes and global objects defined by the library belong to the namespace qpp. To avoid additional typing, we will omit the prefix qpp:: in the rest of this document. We recommend the using of using namespace qpp; in your main .cpp file.

### 3.1 Data types

The most important data types are defined in the header file types.h. We list them in Table 1.

**Table 1:** User-defined data types.

idx	Index (non-negative integer), alias for std::size_t
bigint	Big integer, alias for long long int
cplx	Complex number, alias for std::complex<double>
cmat	Complex dynamic matrix, alias for Eigen::MatrixXcd
dmat	Double dynamic matrix, alias for Eigen::MatrixXd
ket	Complex dynamic column vector, alias for Eigen::VectorXcd
bra	Complex dynamic row vector, alias for Eigen::RowVectorXcd
dyn_mat<Scalar>	Dynamic matrix template alias over the field Scalar, alias for Eigen::Matrix<Scalar, Eigen::Dynamic, Eigen::Dynamic>
dyn_col_vect<Scalar>	Dynamic column vector template alias over the field Scalar, alias for Eigen::Matrix<Scalar, Eigen::Dynamic, 1>
dyn_row_vect<Scalar>	Dynamic row vector template alias over the field Scalar, alias for Eigen::Matrix<Scalar, 1, Eigen::Dynamic>

### 3.2 Constants

The important constants are defined in the header file constants.h and are listed in Table 2.

**Table 2:** User-defined constants.

constexpr idx maxn = 64;	Maximum number of allowed qu(d)its (subsystems)
constexpr double pi = 3.1415…;	*π*
constexpr double ee = 2.7182…;	*e*, base of natural logarithms
constexpr double eps = 1e-12;	Used in comparing floating point values to zero
constexpr double chop = 1e-10;	Used in display manipulators to set numbers to zero
constexpr double infty = …;	Used to denote infinity in double precision
constexpr cplx operator“”_i (unsigned long long int x)	User-defined literal for the imaginary number $i≔\sqrt{-1}$
constexpr cplx operator“”_i (unsigned long double int x)	User-defined literal for the imaginary number $i≔\sqrt{-1}$
cplx omega(idx D)	*D*-th root of unity *e*^2*πi*/*D*^

### 3.3 Singleton classes and their global instances

Some useful classes are defined as singletons and their instances are globally available, being initialized at runtime in the header file qpp.h, before main(). They are listed in Table 3.

**Table 3:** Global singleton classes and instances.

const Init& init = Init::get_instance();	Library initialization
const Codes& codes = Codes::get_instance();	Quantum error correcting codes
const Gates& gt = Gates::get_instance();	Quantum gates
const States& st = States::get_instance();	Quantum states
RandomDevices& rdevs = RandomDevices::get_thread_local_instance();	Random devices/generators/engines

## 4 Simple examples

All of the examples of this section are copied verbatim from the directory ./examples and are fully compilable. For convenience, the location of the source file is displayed in the first line of each example as a C++ comment. The examples are simple and demonstrate the main features of Quantum++. They cover only a small part of library functions, but enough to get the interested user started. For an extensive reference of all library functions, including various overloads, the user should consult the complete reference ./doc/refman.pdf. See also the rest of the examples (not discussed in this document) in ./examples/. for more comprehensive code snippets.

### 4.1 Gates and states

Let us introduce the main objects used by Quantum++: gates, states and basic operations. Consider the code in Listing 2.

**Listing 2. Gates and states**

1 // Gates and states

2 // Source: ./examples/gates_states.cpp

3 #include <iostream>

4

5 #include “qpp.h”

6

7 int main() {

8  using namespace qpp;

9  ket psi = st.z0; // |0> state

10  cmat U = gt.X;

11  ket result = U * psi;

12

13  std::cout << “>> The result of applying the bit-flip gate X on |0> is:\n”;

14  std::cout << disp(result) << ‘\n’;

15

16  psi = 10_ket;   // |10> state

17  U = gt.CNOT;  // Controlled-NOT

18  result = U * psi;

19

20  std::cout << “>> The result of applying the gate CNOT on |10> is:\n”;

21  std::cout << disp(result) << ‘\n’;

22

23  U = randU();

24  std::cout << “>> Generating a random one-qubit gate U:\n”;

25  std::cout << disp(U) << ‘\n’;

26

27  result = applyCTRL(psi, U, {0}, {1}); // Controlled-U

28  std::cout << “>> The result of applying the CTRL-U gate on |10> is:\n”;

29  std::cout << disp(result) << ‘\n’;

30 }

A possible output is:

**Listing 2 output**

>> The result of applying the bit-flip gate X on |0> is:

0

1

>> The result of applying the gate CNOT on |10> is:

0

0

0

1

>> Generating a random one-qubit gate U:

-0.251227 − 0.849866i -0.0204441 − 0.462811i

-0.0716251 + 0.457692i 0.343895 − 0.816777i

>> The result of applying the CTRL-U gate on |10> is:

        0

        0

-0.251227 − 0.849866i

-0.0716251 + 0.457692i

In line 5 of Listing 2 we bring the namespace qpp into the global namespace.

In line 9 we use the States singleton st to declare psi as the zero eigenvector |0〉 of the *Z* Pauli operator. In line 10 we use the Gates singleton gt and assign to U the bit flip gate gt.X. In line 11 we compute the result of the operation *X*|0〉, and display the result |1〉 in lines 13 and 14. In line 14 we use the display manipulator disp(), which is especially useful when displaying complex matrices, as it displays the entries of the latter in the form *a* + *bi*, in contrast to the form (*a*, *b*) used by the C++ standard library. The manipulator also accepts additional parameters that allows e.g. setting to zero numbers smaller than some given value (useful to chop small values), and it is in addition overloaded for standard containers, iterators and C-style arrays.

In line 16 we reassign to psi the state |10〉 via the user-defined literal ket operator“” _ket(). We could have also used the Eigen 3 insertion operator

ket psi(4); // specify the dimension before insertion of elements via <<

psi << 0, 0, 1, 0;

or the Quantum++ library function mket(). In line 17 we declare a gate U as the Controlled-NOT with control as the first subsystem, and target as the last, using the global singleton gt. In line 18 we declare the ket result as the result of applying the Controlled-NOT gate to the state |10〉, i.e. |11〉. We then display the result of the computation in lines 20 and 21.

Next, in line 23 we generate a random unitary gate via the function randU(), then in line 27 apply the Controlled-U, with control as the first qubit and target as the second qubit, to the state psi. Finally, we display the result in lines 28 and 29.

### 4.2 Measurements

Let us now complicate things a bit and introduce measurements. Consider the example in Listing 3.

**Listing 3. Measurements**

1 // Measurements

2 // Source: ./examples/measurements.cpp

3 #include <iostream>

4 #include <tuple>

5

6 #include “qpp.h”

7

8 int main() {

9  using namespace qpp;

10  ket psi = 00_ket;

11  cmat U = gt.CNOT * kron(gt.H, gt.Id2);

12  ket result = U * psi; // we have the Bell state (|00> + |11>) / sqrt(2)

13

14  std::cout << “>> We just produced the Bell state:\n”;

15  std::cout << disp(result) << ‘\n’;

16

17  // apply a bit flip on the second qubit

18  result = apply(result, gt.X, {1}); // we produced (|01> + |10>) / sqrt(2)

19  std::cout << “>> We produced the Bell state:\n”;

20  std::cout << disp(result) << ‘\n’;

21

22  // measure the first qubit in the X basis

23  auto measured = measure(result, gt.H, {0});

24  std::cout << “>> Measurement result: “<< std::get<0>(measured) << ‘\n’;

25  std::cout << “>> Probabilities: “;

26  std::cout << disp(std::get<1>(measured), “,”) << ‘\n’;

27  std::cout << “>> Resulting states: \n”;

28  for (auto&& it: std::get<2>(measured))

29   std::cout << disp(it) << “\n\n”;

30 }

A possible output is:

**Listing 3 output**

>> We just produced the Bell state:

0.707107

   0

   0

0.707107

>> We produced the Bell state:

   0

0.707107

0.707107

   0

>> Measurement result: 1

>> Probabilities: [0.5, 0.5]

>> Resulting states:

0.707107

0.707107

-0.707107

0.707107

In line 11 of Listing 3 we use the function kron() to create the tensor product (Kronecker product) of the Hadamard gate on the first qubit and identity on the second qubit, then we left-multiply it by the Controlled-NOT gate. In line 12 we compute the result of the operation *CNOT*_*ab*_(*H* ⊗ *I*)|00〉, which is the Bell state $(|00\rangle +\left|11\rangle \right)/\sqrt{2}$. We display it in lines 14 and 15.

In line 18 we use the function apply() to apply the gate *X* on the second qubit of the previously produced Bell state. Note that Quantum++ uses the C/C++ numbering convention, with indexes starting from zero. The function apply() takes as its third parameter a list of subsystems, and in our case {1} denotes the second subsystem, not the first. The function apply(), as well as many other functions that we will encounter, have a variety of useful overloads, see doc/refman.pdf for a detailed library reference. In lines 19 and 20 we display the newly created Bell state.

In line 23 we use the function measure() to perform a measurement of the first qubit (subsystem {0}) in the *X* basis. You may be confused by the apparition of gt.H, however this overload of the function measure() takes as its second parameter the measurement basis, specified as the columns of a complex matrix. In our case, the eigenvectors of the *X* operator are just the columns of the Hadamard matrix. As mentioned before, as all other library functions, measure() returns by value, hence it does not modify its argument. The return of measure is a tuple consisting of the measurement result, the outcome probabilities, and the possible output states. Technically measure() returns a tuple of 3 elements

std::tuple<qpp::idx, std::vector<double>, std::vector<qpp::cmat>>

The first element represents the measurement result, the second the possible output probabilities and the third the output states. Instead of using this cumbersome type definition, we use the new C++11 auto keyword to infer the type of the result measured returned by measure(). In lines 24–29 we use the standard std::get<>() function to retrieve each element of the tuple, then display the measurement result, the probabilities and the resulting output states.

### 4.3 Quantum operations

In Listing 4 we introduce quantum operations: quantum channels, as well as the partial trace and partial transpose operations.

**Listing 4. Quantum operations**

1 // Quantum operations

2 // Source: ./examples/quantum_operations.cpp

3 #include <iostream>

4 #include <vector>

5

6 #include “qpp.h”

7

8 int main() {

9  using namespace qpp;

10  cmat rho = st.pb00; // projector onto the Bell state (|00> + |11>) / sqrt(2)

11  std::cout << “>> Initial state:\n”;

12  std::cout << disp(rho) << ‘\n’;

13

14  // partial transpose of first subsystem

15  cmat rhoTA = ptranspose(rho, {0});

16  std::cout << “>> Eigenvalues of the partial transpose”

17      << “of Bell-0 state are:\n”;

18  std::cout << disp(transpose(hevals(rhoTA))) << ‘\n’;

19

20  std::cout << “>> Measurement channel with 2 Kraus operators:\n”;

21  std::vector<cmat> Ks{st.pz0, st.pz1}; // 2 Kraus operators

22  std::cout << disp(Ks[0]) << “\nand\n” << disp(Ks[1]) << ‘\n’;

23

24  std::cout << “>> Superoperator matrix of the channel:\n”;

25  std::cout << disp(kraus2super(Ks)) << ‘\n’;

26

27  std::cout << “>> Choi matrix of the channel:\n”;

28  std::cout << disp(kraus2choi(Ks)) << ‘\n’;

29

30  // apply the channel onto the first subsystem

31  cmat rhoOut = apply(rho, Ks, {0});

32  std::cout << “>> After applying the measurement channel”

33      << “on the first qubit:\n”;

34  std::cout << disp(rhoOut) << ‘\n’;

35

36  // take the partial trace over the second subsystem

37  cmat rhoA = ptrace(rhoOut, {1});

38  std::cout << “>> After partially tracing down the second subsystem:\n”;

39  std::cout << disp(rhoA) << ‘\n’;

40

41  // compute the von-Neumann entropy

42  double ent = entropy(rhoA);

43  std::cout << “>> Entropy: “<< ent << ‘\n’;

44 }

The output of this program is:

**Listing 4 output**

>> Initial state:

0.5 0 0 0.5

 0 0 0 0

 0 0 0 0

0.5 0 0 0.5

>> Eigenvalues of the partial transpose of Bell-0 state are:

-0.5 0.5 0.5 0.5

>> Measurement channel with 2 Kraus operators:

1 0

0 0

and

0 0

0 1

>> Superoperator matrix of the channel:

1 0 0 0

0 0 0 0

0 0 0 0

0 0 0 1

>> Choi matrix of the channel:

1 0 0 0

0 0 0 0

0 0 0 0

0 0 0 1

>> After applying the measurement channel on the first qubit:

0.5 0 0 0

 0 0 0 0

 0 0 0 0

 0 0 0 0.5

>> After partially tracing down the second subsystem:

0.5  0

 0 0.5

>> Entropy: 1

The example should by now be self-explanatory. In line 10 of Listing 4 we define the input state rho as the projector onto the Bell state $(|00\rangle +\left|11\rangle \right)/\sqrt{2}$, then display it in lines 11 and 12.

In lines 15–18 we partially transpose the first qubit, then display the eigenvalues of the resulting matrix rhoTA.

In lines 20–22 we define a quantum channel Ks consisting of two Kraus operators: |0〉〈0| and |1〉〈1|, then display the latter. Note that Quantum++ uses the std::vector<cmat> container to store the Kraus operators and define a quantum channel.

In lines 24–28 we display the superoperator matrix as well as the Choi matrix of the channel Ks.

Next, in lines 31–34 we apply the channel Ks to the first qubit of the input state rho, then display the output state rhoOut.

In lines 37–39 we take the partial trace of the output state rhoOut, then display the resulting state rhoA.

Finally, in lines 42 and 43 we compute the von-Neumann entropy of the resulting state and display it.

### 4.4 Timing

To facilitate simple timing tasks, Quantum++ provides a Timer<> class that uses internally a std::steady_clock. The program in Listing 5 demonstrate its usage.

**Listing 5. Timing**

1 // Timing

2 // Source: ./examples/timing.cpp

3 #include <iomanip>

4 #include <iostream>

5 #include <vector>

6

7 #include “qpp.h”

8

9 int main() {

10  using namespace qpp;

11  std::cout << std::setprecision(8); // increase the default output precision

12

13  // get the first codeword from Shor’s [[9,1,3]] code

14  ket c0 = codes.codeword(Codes::Type::NINE_QUBIT_SHOR, 0);

15

16  Timer<> t;             // declare and start a timer

17  std::vector<idx> perm = randperm(9); // declare a random permutation

18  ket c0perm = syspermute(c0, perm);  // permute the system

19  t.toc();              // stops the timer

20  std::cout << “>> Permuting subsystems according to “<< disp(perm, “, “);

21  std::cout << “\n>> It took “<< t << “seconds to permute the subsytems. \n”;

22

23  t.tic();   // restart the timer

24  std::cout << “>> Inverse permutation: ”;

25  std::cout << disp(invperm(perm), “, “) << ‘\n’;

26  ket c0invperm = syspermute(c0perm, invperm(perm)); // permute again

27  std::cout << “>> It took “<< t.toc();

28  std::cout << “seconds to un-permute the subsystems. \n”;

29

30  std::cout << “>> Norm difference: “<< norm(c0invperm − c0) << ‘\n’;

31 }

A possible output of this program is:

**Listing 5 output**

>> Permuting subsystems according to [7, 5, 3, 4, 2, 6, 0, 8, 1]

>> It took 0.000161381 seconds to permute the subsytems.

>> Inverse permutation: [6, 8, 4, 2, 3, 1, 5, 0, 7]

>> It took 0.000104443 seconds to un-permute the subsystems.

>> Norm difference: 0

In line 11 of Listing 5 we change the default output precision from 4 to 8 decimals after the delimiter.

In line 14 we use the Codes singleton codes to retrieve in c0 the first codeword of the Shor’s [[9, 1, 3]] quantum error correcting code.

In line 16 we declare an instance timer of the class Timer<>. In line 17 we declare a random permutation perm via the function randperm(). In line 18 we permute the codeword according to the permutation perm using the function syspermute() and store the result. In line 19 we stop the timer. In line 20 we display the permutation, using an overloaded form of the disp() manipulator for C++ standard library containers. The latter takes a std::string as its second parameter to specify the delimiter between the elements of the container. In line 21 we display the elapsed time using the ostream operator<<() operator overload for Timer<> instances.

Next, in line 23 we reset the timer, then display the inverse permutation of perm in lines 24 and 25. In line 26 we permute the already permuted state c0perm according to the inverse permutation of perm, and store the result in c0invperm. In lines 27 and 28 we display the elapsed time. Note that in line 27 we used directly t.toc() in the stream insertion operator, since, for convenience, the member function Timer<>::toc() returns a const Timer<>&.

Finally, in line 30, we verify that by permuting and permuting again using the inverse permutation we recover the initial codeword, i.e. the norm difference has to be zero.

### 4.5 Input/Output

We now introduce the input/output functions of Quantum++, as well as the input/output interfacing with MATLAB. The program in Listing 6 saves a matrix in both Quantum++ internal format as well as in MATLAB format, then loads it back and tests that the norm difference between the saved/loaded matrix is zero.

**Listing 6. Input/output**

1 // Input/output

2 // Source: ./examples/input_output.cpp

3 #include <iostream>

4

5 #include “qpp.h”

6 #include “MATLAB/matlab.h” // must be explicitly included

7

8 int main() {

9  using namespace qpp;

10  // Quantum++ native input/output

11  cmat rho = randrho(256);        // an 8 qubit density operator

12  save(rho, “rho.dat”);          // save it

13  cmat loaded_rho = load<cmat>(“rho.dat”); // load it back

14  // display the difference in norm, should be 0

15  std::cout << “>> Norm difference load/save: “;

16  std::cout << norm(loaded_rho − rho) << ‘\n’;

17

18  // interfacing with MATLAB

19  saveMATLAB(rho, “rho.mat”, “rho”, “w”);

20  loaded_rho = loadMATLAB<cmat>(“rho.mat”, “rho”);

21  // display the difference in norm, should be 0

22  std::cout << “>> Norm difference MATLAB load/save:”;

23  std::cout << norm(loaded_rho − rho) << ‘\n’;

24 }

The output of this program is:

**Listing 6 output**

>> Norm difference load/save: 0

>> Norm difference MATLAB load/save: 0

Note that in order to use the MATLAB input/output interface support, you need to explicitly include the header file MATLAB/matlab.h, and you also need to have MATLAB or MATLAB compiler installed, otherwise the program fails to compile. See the Wiki for extensive details about compiling with MATLAB support.

### 4.6 Qudit teleportation

As mentioned before, Quantum++ treats qubits and qudits on the same footing. Below is a relatively more advanced self- documented example that implements the teleportation protocol for qudits.

**Listing 7. Qudit teleporation**

1 // Qudit teleporation

2 // Source: ./examples/teleport_qudit.cpp

3 #include <cmath>

4 #include <iostream>

5 #include <tuple>

6 #include <vector>

7

8 #include “qpp.h”

9

10 int main() {

11  using namespace qpp;

12  idx D = 3; // size of the system

13  std::cout << “>> Qudit teleportation, D = “<< D << ‘\n’;

14

15  ket mes_AB = st.mes(D); // maximally entangled state resource

16

17  // circuit that measures in the qudit Bell basis

18  cmat Bell_aA =

19   adjoint(gt.CTRL(gt.Xd(D), {0}, {1}, 2, D) * kron(gt.Fd(D), gt.Id(D)));

20

21  ket psi_a = randket(D); // random qudit state

22  std::cout << “>> Initial state:\n”;

23  std::cout << disp(psi_a) << ‘\n’;

24

25  ket input_aAB = kron(psi_a, mes_AB); // joint input state aAB

26  // output before measurement

27  ket output_aAB = apply(input_aAB, Bell_aA, {0, 1}, D);

28

29  // measure on aA

30  auto measured_aA = measure(output_aAB, gt.Id(D * D), {0, 1}, D);

31  idx m = std::get<0>(measured_aA); // measurement result

32

33  std::vector<idx> midx = n2multiidx(m, {D, D});

34  std::cout << “>> Alice measurement result: “;

35  std::cout << m << “-> “<< disp(midx, “”) << ‘\n’;

36  std::cout << “>> Alice measurement probabilities: “;

37  std::cout << disp(std::get<1>(measured_aA), “, “) << ‘\n’;

38

39  // conditional result on B before correction

40  ket output_m_B = std::get<2>(measured_aA)[m];

41

42  // perform the correction on B

43  cmat correction_B =

44   powm(gt.Zd(D), midx[0]) * powm(adjoint(gt.Xd(D)), midx[1]);

45  std::cout << “>> Bob must apply the correction operator Z^“<< midx[0]

46     << “X^” << (D − midx[1]) % D << ‘\n’;

47  ket psi_B = correction_B * output_m_B;

48

49  // display the output

50  std::cout << “>> Bob final state (after correction): \n”;

51  std::cout << disp(psi_B) << ‘\n’;

52

53  // verification

54  std::cout << “>> Norm difference: “<< norm(psi_B − psi_a) << ‘\n’;

55 }

The output of this program is:

**Listing 7 output**

>> Qudit teleportation, D = 3

>> Initial state:

0.305468 + 0.0132564i

-0.274931 − 0.690466i

-0.537024 − 0.256493i

>> Alice measurement result: 2 -> [0 2]

>> Alice measurement probabilities: [0.111111, 0.111111, 0.111111, 0.111111,

 0.111111, 0.111111, 0.111111, 0.111111, 0.111111]

>> Bob must apply the correction operator Z^0 X^1

>> Bob final state (after correction):

0.305468 + 0.0132564i

-0.274931 − 0.690466i

-0.537024 − 0.256493i

>> Norm difference: 1.23512e-15

### 4.7 Exceptions

Most Quantum++ functions throw exceptions in the case of unrecoverable errors, such as out-of-range input parameters, input/output errors etc. The exceptions are handled via the class Exception, derived from std::exception. The exception types are hard-coded inside the strongly-typed enumeration (enum class) Exception::Type. If you want to add more exceptions, augment the enumeration Exception::Type and also modify accordingly the member function Exception::construct_exception_msg_(), which constructs the exception message displayed via the overridden virtual function Exception::what(). Listing 8 illustrates the basics of exception handling in Quantum++.

**Listing 8. Exceptions**

1 // Exceptions

2 // Source: ./examples/exceptions.cpp

3 #include <exception>

4 #include <iostream>

5

6 #include “qpp.h”

7

8 int main() {

9  using namespace qpp;

10  cmat rho = randrho(16); // 4 qubits (subsystems)

11  try {

12    // the line below throws qpp::exception::SubsysMismatchDims

13    double mInfo = qmutualinfo(rho, {0}, {4});

14    std::cout << “>> Mutual information between first and last subsystem: “;

15    std::cout << mInfo << ‘\n’;

16  } catch (const std::exception& e) {

17    std::cout << “>> Exception caught: “<< e.what() << ‘\n’;

18  }

19 }

The output of this program is:

**Listing 8 output**

>> Exception caught: IN qpp::qmutualinfo(): Subsystems mismatch dimensions!

In line 10 of Listing 8 we define a random density matrix on four qubits (dimension 16). In line 13, we compute the mutual information between the first and the 5-th subsystem (which does not exist). Line 13 throws an exception of type qpp::exception::SubsysMismatchDim exception, as there are only four systems. We next catch the exception in line 16 via the std::exception standard exception base class. We could have also used the Quantum++ exception base class qpp::exception::Exception, however using the std::exception allows the catching of other exceptions, not just of the type Exception. Finally, in line 17 we display the corresponding exception message.

### 4.8 Classical reversible logic

Quantum++ provides support for classical reversible logic and circuits via two classes, Dynamic_bitset and Bit_circuit. The first is similar to the standard library std::bitset, with the exception that the length of the bitset can be specified at runtime, whereas the latter is used to describe a classical reversible bit circuit and provides the required interface for applying gates, retrieving bit values etc. The example in Listing 9 is self-explanatory.

**Listing 9. Classical reversible logic**

1 // Reversible classical circuits

2 // Source: ./examples/reversible.cpp

3 #include <iostream>

4

5 #include “qpp.h”

6

7 int main() {

8  using namespace qpp;

9  std::cout << “>> Classical reversible circuits. “;

10  std::cout << “Bits are labeled from right to left,\n “;

11  std::cout << “i.e. bit zero is the least significant bit (rightmost).\n”;

12

13  Dynamic_bitset bits{4};            // 4 classical bits

14  std::cout << “>> Initial bitset:\n\t” << bits << ‘\n’; // display them

15

16  bits.rand(); // randomize the bits

17  std::cout << “>> After randomization:\n\t” << bits << ‘\n’; // display them

18

19  Bit_circuit bit_circuit{bits}; // bit circuit

20

21  std::cout << “>> Apply X_0, followed by CNOT_02, CNOT_13 and TOF_013\n”;

22  bit_circuit.X(0); // apply a NOT gate on first bit

23  bit_circuit.CNOT({0, 2}).CNOT({1, 3}).TOF({0, 1, 3}); // sequence operations

24

25  std::cout << “>> Final bit circuit:\n\t” << bit_circuit << ‘\n’;

26  std::cout << “>> 3rd bit: “<< bit_circuit.get(2) << ‘\n’;

27  std::cout << “>> CNOT count: “<< bit_circuit.gate_count.CNOT << ‘\n’;

28

29  bit_circuit.reset(); // resets the circuit

30  std::cout << “>> Reseted circuit:\n\t” << bit_circuit << ‘\n’;

31  std::cout << “>> CNOT count: “<< bit_circuit.gate_count.CNOT << ‘\n’;

32 }

The output of this program is:

**Listing 9 output**

>> Classical reversible circuits. Bits are labeled from right to left,

 i.e. bit zero is the least significant bit (rightmost).

>> Initial bitset:

  0000

>> After randomization:

  0110

>> Apply X_0, followed by CNOT_02, CNOT_13 and TOF_013

>> Final bit circuit:

  0011

>> 3rd bit: 0

>> CNOT count: 2

>> Reseted circuit:

  0000

>> CNOT count: 0

## 5 Advanced topics

### 5.1 Aliasing

Aliasing occurs whenever the same Eigen 3 matrix/vector appears on both sides of the assignment operator, and happens because of Eigen 3’s lazy evaluation system. Examples that exhibit aliasing:

mat = 2 * mat;

or

mat = mat.transpose();

Aliasing does not occur in statements like

mat = f(mat);

where f() returns by value. Aliasing produces in general unexpected results, and should be avoided at all costs.

Whereas the first line produces aliasing, it is not dangerous, since the assignment is done in a one-to-one manner, i.e. each element (*i*, *j*) on the left hand side of the assignment operator is solely a function of the the same (*i*, *j*) element on the right hand side, i.e. *mat*(*i*, *j*) = *f*(*mat*(*i*, *j*)), ∀*i*, *j*. The problem appears whenever coefficients are being combined and overlap, such as in the second example, where *mat*(*i*, *j*) = *mat*(*j*, *i*), ∀*i*, *j*. To avoid aliasing, use the member function eval() to transform the right hand side object into a temporary, such as

mat = 2 * mat.eval();

In general, aliasing can not be detected at compile time, but can be detected at runtime whenever the compile flag EIGEN_NO_DEBUG is not set. Quantum++ does not set this flag in debug mode. We highly recommend to first compile your program in debug mode to detect aliasing run-time assertions, as well as other possible issues that may have escaped you, such as assigning a matrix to another matrix of mismatching dimensions etc.

For more details about aliasing, see the official Eigen 3 documentation at http://eigen.tuxfamily.org/dox/group__TopicAliasing.html.

### 5.2 Type deduction via auto

Avoid the usage of auto when working with Eigen 3 expressions, e.g. avoid writing code like

auto mat = A * B + C;

but write instead

cmat mat = A * B + C;

or

auto mat = (A * B + C).eval();

to force evaluation, as otherwise you may get unexpected results. The “problem” lies in the Eigen 3 lazy evaluation system and reference binding, see e.g. http://stackoverflow.com/q/26705446/3093378 for more details. In short, the reference to the internal data represented by the expression A * B + C is dangling at the end of the auto mat = A * B + C; statement.

### 5.3 Optimizations

Whenever testing your application, we recommend compiling in debug mode, as Eigen 3 run-time assertions can provide extremely helpful feedback on potential issues. Whenever the code is production-ready, you should always compile with optimization flags turned on, such as -O3 (for g++) and -DEIGEN_NO_DEBUG. You should also turn on the OpenMP (if available) multi-processing flag (-fopenmp for g++), as it enables multi-core/multi-processing with shared memory. Eigen 3 uses multi-processing when available, e.g. in matrix multiplication. Quantum++ also uses multi-processing in computationally-intensive functions.

Since most Quantum++ functions return by value, in assignments of the form

mat = f(another_mat);

there is an additional copy assignment operator when assigning the temporary returned by f() back to mat. As far as we are aware, this extra copy operation is not elided. Unfortunately, Eigen 3 does not yet support move semantics, which would have got rid of this additional assignment via the corresponding move assignment operator. If in the future Eigen 3 will support move semantics, the additional assignment operator will be “free”, and you won’t have to modify any existing code to enable the optimization; the Eigen 3 move assignment operator should take care of it for you.

Note that in a line of the form

cmat mat = f(another_mat);

most compilers perform return value optimization (RVO), i.e. the temporary on the right hand side is constructed directly inside the object mat, the copy constructor being elided.

### 5.4 Extending Quantum++

Most Quantum++ operate on Eigen 3 matrices/vectors, and return either a matrix or a scalar. In principle, you may be tempted to write a new function such as

cmat f(const cmat& A){…}

The problem with the approach above is that Eigen 3 uses expression templates as the type of each expression, i.e. different expressions have in general different types, see the official Eigen 3 documentation at http://eigen.tuxfamily.org/dox/TopicFunctionTakingEigenTypes.html for more details. The correct way to write a generic function that is guaranteed to work with any matrix expression is to make the function template and declare the input parameter as Eigen::MatrixBase<Derived>, where Derived is the template parameter. For example, the Quantum++ transpose() function is defined as

1 template<typename Derived>

2 dyn_mat<typename Derived::Scalar>

3 transpose(const Eigen::MatrixBase<Derived>& A){

4  const dyn_mat<typename Derived::Scalar>& rA = A.derived();

5

6  // check zero-size

7  if (!internal::check_nonzero_size(rA))

8   throw Exception(“qpp::transpose()”, Exception::Type::ZERO_SIZE);

9

10  return rA.transpose();

11 }

It takes an Eigen 3 matrix expression, line 3, and returns a dynamic matrix over the scalar field of the expression, line 2. In line 4 we implicitly convert the input expression A to a dynamic matrix rA over the same scalar field as the expression, via binding to a const reference, therefore paying no copying cost. We then use rA instead of the original expression A in the rest of the function. Note that most of the time it is adequate to use the original expression, however there are some cases where you may get a compile time error if the expression is not explicitly cast to a matrix. For consistency, we use this reference binding trick in the code of all Quantum++ functions.

## 6 Benchmarks

In this section we compare the performance of Quantum++ with two other widely used quantum software platforms that allow quantum simulation, namely IBM’s Qiskit and the open source QuTiP. More specifically, we benchmark the time required to perform two widely used quantum operations, namely the partial trace and the quantum Fourier transform, respectively, as a function of the number of input qubits and number of CPU cores. Note that both vanilla versions of Qiskit and QuTiP do not seem to be using parallelization, so for a fair comparison the reader should only compare with the single-threaded curve(s) generated for Quantum++. All benchmark plots use logarithmic (base 2) scales for the time scale (expressed in seconds). We used an 8 core x86-64 Linux machine running Debian 9.5, with an Intel(R) Core(TM) i7-7700K CPU running at 4.20GHz and 16Gb of RAM. Quantum++ was compiled with g++ 6.3, whereas the Qiskit and QuTiP simulators were run using Python 3.5. All benchmark code from this section is available online at https://github.com/vsoftco/qpp/tree/master/stress_tests.

### 6.1 Partial trace

**Fig 1:**
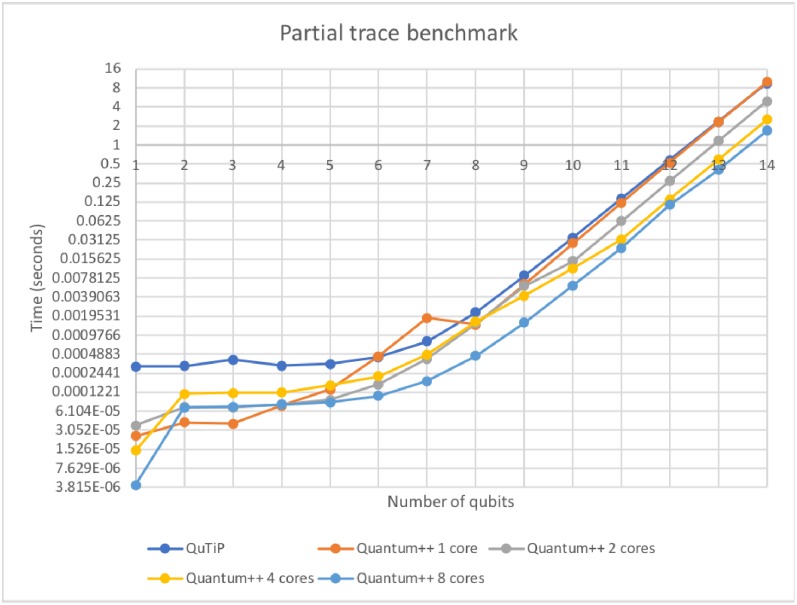
Partial trace on *n* qubits. Some minor irregularities (spikes) in the plot are most likely due to the fact that the machine we ran the experiments on is not real-time, and the operating system may have performed job scheduling during that time. Note that the single core version of Quantum++ over-performs QuTiP. Remark also that Quantum++ scales well with the number of CPU cores.

In this subsection we benchmark how long it takes to perform a partial trace over the first qubit of an *n*-qubit matrix. Note that Qiskit does not provide a native partial trace function, so we only benchmark against QuTiP. The results are displayed in Fig 1.

### 6.2 Quantum Fourier transform

**Fig 2:**
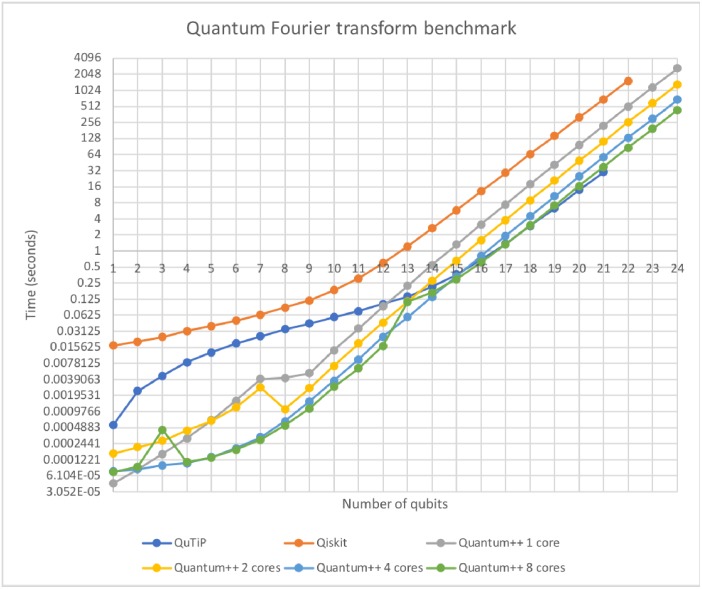
Quantum Fourier transform on *n* qubits. Some minor irregularities (spikes) in the plot are most likely due to the fact that the machine we ran the experiments is not real-time, and the operating system may have performed job scheduling during that time. Note that the single core version of Quantum++ over-performs Qiskit by a large margin. QuTiP seems to be faster in this case than Quantum++. The most likely explanation is that the former uses sparse matrices during computation, whereas the latter does not. However, QuTiP runs out of memory on our machine after 21 qubits, whereas Quantum++ can simulate up to 28 qubits without problems (of course trading the space for longer running time). Remark also that Quantum++ scales well with the number of CPU cores.

In this subsection we benchmark how long it takes to perform a quantum Fourier transform over the first qubit of an *n*-qubit matrix. The results are displayed in Fig 2.

### 6.3 Discussion

The fundamental data types in Quantum++ are non-sparse vectors and matrices. Most computationally-intensive activity performed by the library involves operations on such vectors and matrices. Whenever possible, the task is delegated to the highly-optimized Eigen 3 linear algebra library, e.g. when multiplying 2 matrices together. In addition, most loops in the code are parallelized via the OpenMP multi-processing library, if the corresponding flag is present at compile time. Those optimizations make Quantum++ highly efficient on multiple cores, as the benchmarks in Figs 1 and 2 show.

As one of our referees pointed out, QuTiP performs relatively poorly for small number of qubits, most likely because of overhead introduced by the Python interpreter. What is surprising is that both QuTiP and Qiskit seem to be single-cored, even though, at least for QuTiP, one would expect aggressive parallelization via the BLAS library, as mentioned by http://qutip.org/docs/3.0.1/installation.html#optimized-blas-librariesl. Most likely the vanilla version of QuTiP comes with a NumPy library which is not built against BLAS.

## 7 Long term maintenance

We plan to keep all future releases of Quantum++ open source. We will continue to host the project on GitHub or on an equivalent versioning control system. We will publish new stable releases of the software whenever enough improvements or features have been accumulated since the previous stable release. We plan to keep Quantum++ active and we welcome everyone interested to collaborate.

## 8 Conclusions and future directions

As you may have already seen, Quantum++ consists mainly of a collection of functions and few classes. There is no complicated class hierarchy, and you can regard the Quantum++ API as a low/medium-level API. You may extend it to incorporate graphical input, e.g. use a graphical library such as Qt, or build a more sophisticated library on top of it. We recommend to read the source code and make yourself familiar with the library before deciding to extend it. You should also check the complete reference manual ./doc/refman.pdf for an extensive documentation of all functions and classes.

An interesting future direction is to allow GPU parallelization, however at the time of the writing this was beyond the scope of this project.
